# Perceived insufficient milk among primiparous, fully breastfeeding women: Is infant crying important?

**DOI:** 10.1111/mcn.13133

**Published:** 2021-01-05

**Authors:** Lisa M. Mohebati, Peter Hilpert, Sarah Bath, Margaret P. Rayman, Monique M. Raats, Homero Martinez, Laura E. Caulfield

**Affiliations:** ^1^ Center for Human Nutrition, Department of International Health Johns Hopkins University Bloomberg School of Public Health Baltimore Maryland USA; ^2^ School of Psychology University of Surrey Guildford Surrey UK; ^3^ Nutrition, Food and Exercise Sciences Department, School of Biosciences and Medicine University of Surrey Guildford Surrey UK; ^4^ Food, Consumer Behaviour and Health Research Centre University of Surrey Guildford Surrey UK; ^5^ NTEAM Nutrition International Ottawa Ontario Canada; ^6^ Hospital Infantil de México Federico Gómez Mexico City Mexico

**Keywords:** breast feeding, crying, infant, infant care, lactation, milk, mothers

## Abstract

Breastfeeding mothers often report perceived insufficient milk (PIM) believing their infant is crying too much, which leads to introducing formula and the early abandonment of breastfeeding. We sought to determine if infant crying was associated with reported PIM (yes/no) and number of problems associated with lactation (lactation problem score [LPS] 6‐point Likert scale) before formula introduction. Primiparous breastfeeding mothers were recruited at birth and visited at 1, 2 and 4 weeks. Among those fully breastfeeding at 1 week (*N* = 230), infant crying variables based on maternal reports were not associated with PIM at 1 week, but LPS was. However, a mother's expectation that her infant would cry more than other infants was associated with increased odds of reporting PIM at 2 and 4 weeks, as were delayed onset of lactation and previous LPS. At 1 week, crying variables (frequency, difficulty in soothing) were associated with LPS along with percent weight change. Delayed onset of lactation, infant care style, number of breastfeeds and previous LPS were longitudinally associated with change in LPS from 1 to 2 weeks and 2 to 4 weeks. Our data suggest that reported infant crying is associated with PIM and LPS in the first 4 weeks of life. Guidance on what to expect in crying behaviour and the impact of infant care style may be beneficial in reducing PIM and LPS in the first month.

**Key messages**

- Reported infant crying is associated with perceived insufficient milk and reported lactation problems among women fully breastfeeding at 1 and/or 2 weeks of life.
- Higher lactation problem score precedes later reports of perceived insufficient milk, highlighting the need for extra support with lactation as early as possible, especially among women experiencing delayed onset of lactation.
- Giving new mothers anticipatory guidance on crying behaviour and a wider array of coping strategies, especially among mothers who expect it to be challenging for them, has the potential to reduce early lactation problems.

## 1 INTRODUCTION

Exclusive breastfeeding has wide‐reaching benefits to infants, mothers and societies (Victora et al., 2016). However, only about 41% of infants under 6 months of age worldwide are being exclusively breastfed (Global Breastfeeding Collective, 2019). In Mexico, exclusive breastfeeding among infants under 6 months has been reported to be as low as 14.4% in 2012 (Gonzalez de Cossio, Escobar‐Zaragoza, Gonzalez‐Castell, Reyes‐Vazquez, & Rivera‐Dommarco, 2013), translating to a high economic and disease burden to the country (Unar‐Munguia, Stern, Colchero, & Gonzalez de Cosio, 2019). Perceived insufficient milk (PIM) remains at the forefront of the reasons given by women for the introduction of infant formula into the diet of infants under 6 months of age (Perez‐Escamilla, Buccini, Segura‐Perez, & Piwoz, 2019). For the purposes of this study, we defined PIM according to Dykes and Williams' (1999) description of ‘“perceived breast‐milk inadequacy” (…): “A lactating woman's perception that her breast milk supply and/or quality is inadequate for the purposes of exclusively nourishing her baby”’.

Mothers often link infant crying with PIM (Sacco, Caulfield, Gittelsohn, & Martinez, 2006; Segura‐Millan, Dewey, & Perez‐Escamilla, 1994). Crying is an important signal that infants use to indicate hunger. If the infant is breastfed, it is difficult to assess milk intake and crying may lead mothers to believe the amount consumed is not enough, hence leading to PIM. In response, mothers often elect to add formula to the infant's diet (DaMota, Banuelos, Goldbronn, Vera‐Beccera, & Heinig, 2012; Sacco, Caulfield, Gittelsohn, & Martinez, 2006). Some give formula for other reasons, such as inconvenience of or fatigue from breastfeeding (Chang et al., 2019). Previous studies have shown that the introduction of formula to infants who are breastfed is associated with shorter breastfeeding duration (Chantry, Dewey, Peerson, Wagner, & Nommsen‐Rivers, 2014; Forster, McLachlan, & Lumley, 2006; Murray, Ricketts, & Dellaport, 2007; Perez‐Escamilla, Segura‐Millan, Canahuati, & Allen, 1996; Semenic, Loiselle, & Gottlieb, 2008).

Previous studies have examined the effect of feeding on infant crying (Barr & Elias, 1988; Barr, Kramer, Pless, Boisjoly, & Leduc, 1989; Lee, 2000; Lucas & St James‐Roberts, 1998), but we are unaware of studies examining how crying affects feeding choices. Crying has been cited as a reason for breastfeeding cessation (Barr, 1990; Segura‐Millan, Dewey, & Perez‐Escamilla, 1994), and qualitative studies have confirmed associations made by mothers and their relatives between crying and the need to add formula to the infant's diet (Bunik et al., 2006; Sacco, Caulfield, Gittelsohn, & Martinez, 2006; Swigart et al., 2017). What aspect of infant crying (i.e., frequency, difficulty in soothing) is most closely linked to PIM remains unknown.

Breastfeeding difficulties, including PIM, have sometimes been described as a unidimensional problem. There is evidence to suggest PIM may be multifaceted, with different but potentially related causes of a psychological, social or biological nature (Brown, Rance, & Bennett, 2016). For example, maternal anxiety (Dykes & Williams, 1999) and lack of confidence in her parenting (McCarter‐Spaulding & Kearney, 2001) and/or breastfeeding abilities (Galipeau, Baillot, Trottier, & Lemire, 2018), alongside potential pressure from others to give the infant a bottle with formula (Dykes & Williams, 1999) can lead to PIM. Mothers often believe giving their infant formula is the solution to this problem (Sacco, Caulfield, Gittelsohn, & Martinez, 2006), but this may decrease infant suckling at the breast with a consequent reduction in milk production (Dykes & Williams, 1999). A lack of adequate stimulation of the breast can also occur due to lactation problems such as cracked or painful nipples (Brown, Rance, & Bennett, 2016; Kronborg, Harder, & Hall, 2015), a baby who is sleepy or rejecting the breast or infant latch or sucking problems, leading to decreased milk production (Neifert, 2004). If another milk is not introduced and the breast is adequately stimulated, PIM may be limited to a perception, rather than a self‐fulfilling prophecy. This is suggested by a study which showed no significant association between PIM and 24‐h milk production at week 2 (Galipeau, Dumas, & Lepage, 2017).

Delayed onset of lactation (DOL), a delay of more than 72 h postpartum in production of copious breast milk after colostrum, is also a risk factor for PIM (Perez‐Escamilla, Buccini, Segura‐Perez, & Piwoz, 2019; Segura‐Millan, Dewey, & Perez‐Escamilla, 1994). Other factors associated with DOL, such as obesity or delivery by caesarean, are likewise linked to early cessation of exclusive breastfeeding (Brownell, Howard, Lawrence, & Dozier, 2012). Decreased breast milk sodium is a marker for the onset of lactation and has been associated with increased breastfeeding frequency (Galipeau, Goulet, & Chagnon, 2012). Decreased breastfeeding frequency is therefore associated both with DOL as well as PIM (Galipeau, Dumas, & Lepage, 2017) and cannot be ignored in a more detailed exploration of PIM.

Mothers who align themselves with certain infant care styles might also be more concerned by crying behaviour than others. Some mothers adopt a *proximal* infant care style, where on‐demand feeding, responsiveness to the infant's cues and close physical contact during the day and night is predominant, whereas others value a *distal* style with schedules, leaving the baby to self‐soothe and having less physical contact with the infant (Little, Legare, & Carver, 2018). Proximal styles are associated with less crying and greater likelihood of breastfeeding, whereas the opposite is found for distal styles (Brown & Arnott, 2014; Little, Legare, & Carver, 2018; St James‐Roberts et al., 2006). Attempting to schedule or limit feeds has also been associated with PIM (Brown, Raynor, & Lee, 2011). These studies assessed infant care style at a single time‐point across a range of infant ages (0 to 24 months) and examined cross‐sectional associations between infant care style and breastfeeding outcomes. The exception was the study by St James‐Roberts et al. (2006), which assessed infant care style initially and followed study participants until 14 weeks of age.

Our aims were to explore the associations between different aspects of infant crying, PIM and perceived lactation problems among mothers fully breastfeeding according to the definition by Labbok and Krasovec (1990). This includes women exclusively (no other liquid or solid given to the infant other than breast milk) or almost exclusively breastfeeding (only vitamins, minerals, water or other ritualistic feeds—such as tea—given in addition to breast milk). Firstly, we aimed to explore whether specific crying variables and PIM were cross‐sectionally associated in the first week, and whether these same crying variables at one visit were longitudinally associated with PIM at a later follow‐up. Secondly, we aimed to explore how these crying variables were associated with perceived lactation problems (lactation problem score [LPS]: ‘How many problems are you experiencing related to breastfeeding?’ on a 6‐point scale, ranging from *none* to *too many*) both cross‐sectionally (at 1 week) and longitudinally within the first month of life.

## 2 METHODS

### 2.1 Design and procedures

This is part of a larger longitudinal study of mother‐infant dyads followed from birth to 24 weeks of infant age (see also, Mohebati, Caulfield, & Martinez, 2014). Four hundred seventy‐five women were recruited to the study by approaching them in the postpartum ward of an Instituto Mexicano del Seguro Social (IMSS) Baby‐Friendly hospital in Mexico City, Mexico, from March 2000 to May 2002. This hospital was chosen as it was part of the IMSS system at which one of the coinvestigators was based, it had a sufficiently large yearly number of births for the sample size needed and, being Baby‐Friendly, would assist us in recruiting women who were more likely to have received support and encouragement to breastfeed. Despite the passage of time, the study results are still relevant today as PIM continues to be one of the main reasons given by women for the abandonment of full breastfeeding in the first 6 months of life (Perez‐Escamilla, Buccini, Segura‐Perez, & Piwoz, 2019).

Inclusion criteria for the study included being first‐time mothers of singleton, healthy (not having been admitted into the intensive care unit and having been discharged from the hospital within the first week), full‐term (≥37 weeks of gestation) infants who planned to breastfeed their child and not undertake paid employment until the infant's 6 month birth day. This is because we did not want to include women who planned to stop breastfeeding or introduce formula because they were returning to work. Ethical approval was obtained from the relevant institutions in the United Kingdom, the United States and Mexico.

Trained project field workers collected data related to the baby's measurements and delivery from the women's medical charts at the time women gave informed written consent to participate in the study. Women were weighed and measured by project fieldworkers at the hospital and arrangements were made to visit them in their homes. Lactation support was provided by hospital nurses prior to hospital discharge. At each home visit, women were interviewed using the questionnaires described below. Infants were weighed to the nearest 10 g (using SECA 345 battery‐powered digital baby‐weighing scales); measurements were taken in duplicate, following published guidelines, and an average weight was used. The full study consisted of nine visits in the first 6 months of life. In this paper, we include data from recruitment in the hospital and at the first three visits (1, 2 and 4 weeks). This is because the first week postpartum is an important time for the initial establishment of lactation (Brownell, Howard, Lawrence, & Dozier, 2012; Galipeau, Goulet, & Chagnon, 2012; Matias, Nommsen‐Rivers, Creed‐Kanashiro, & Dewey, 2010; Perez‐Escamilla, Buccini, Segura‐Perez, & Piwoz, 2019) and over half of women who stop breastfeeding in the first four months do so in the first 5 weeks (Kronborg & Vaeth, 2004). First‐time mothers have also reported feeling unprepared for the early postpartum period and not having realistic expectations of their infant's behaviour at that time (DaMota, Banuelos, Goldbronn, Vera‐Beccera, & Heinig, 2012). The only incentive women received for participating in the study was a VHS copy of the recording of feeding interactions with her infant. Analyses of these recorded interactions are not included in this paper.

### 2.2 Measures

Our outcome variables were derived from questions related to lactation problems. The LPS originated from the question (‘How many problems are you experiencing related to breastfeeding?’ on a 6‐point scale, ranging from *none* to *too many*). This was followed by a request to describe the problems in an open‐ended question (‘Please explain’). If their explanations included concerns related to breast milk adequacy (quantity or quality), these were classified as a report of PIM (yes/no) in a dichotomous variable. Any additional references related to the quantity or quality of breast milk made by the mother on other related questions (e.g., ‘Do any of your infant's behaviours worry you? Please explain’.) were also counted as a report of PIM. PIM and LPS at 1, 2 and 4 weeks were our main outcome variables.

As mentioned above, LPS was an ordinal variable that provided a measure of the perceived number of lactation problems experienced by the mother. Although we did expect and find PIM and LPS to be correlated, we also found that some mothers reporting a lower LPS score (i.e., no problems) did report PIM, and some mothers reporting a higher LPS score (i.e., more problems) did not report PIM. We thus hypothesized that our PIM and LPS measures were measuring different constructs and that crying might be associated with PIM as well as LPS.

Our main explanatory variables of interest were those related to infant crying: (a) general crying expectation, (b) crying frequency, (c) difficulty in soothing and (d) allowing to self‐soothe. We adapted the Crying Patterns Questionnaire (CPQ; St James‐Roberts & Halil, 1991) to accommodate the linguistic and cultural characteristics of the study sample. Crying expectation was determined from the question at recruitment: ‘Compared to other babies, how much do you think your infant will cry?’; the responses were on a six‐point scale and could range from ‘nearly not at all’ to ‘too much’. We included this question because we were interested in how maternal expectations of infant crying behaviour were associated with reported crying behaviour (Mohebati, Caulfield, & Martinez, 2014). Crying frequency (number of times cried/fussed per day) was determined from the follow‐up visits where mothers were asked how many times and for how long their infant cried and fussed during four defined 6‐h periods over a typical day of the past week. Difficulty in soothing was estimated from the question ‘How easily can you soothe your baby when s/he is crying?’ (a 6‐point scale ranging from *very easily* to *with great difficulty*) and allowing to self‐soothe was estimated from the question ‘How often do you let your baby cry alone until s/he soothes her/himself?’ (a 6‐point scale ranging from *never* to *always*) answered by mothers at each follow‐up visit.

*Distal/proximal* approaches to infant care were determined using the Responsiveness to Crying, Schedules and Infant Satisfaction with Breast Milk questionnaire (RCSISBM). This 39‐item questionnaire had been developed during formative qualitative research in this population and is included in its original format in the Supporting Information. It was completed at 1 week of infant age. It included items such as: ‘One must not immediately go to the baby when s/he is crying, so that s/he can learn to be patient’; ‘For a baby to be satisfied, one must give breast milk and formula’ and ‘When a baby cries frequently during the night it is because breast milk does not fill him/her up’; answers were on a 6‐point scale and ranged from *completely disagree* to *completely agree*. A total percent score was calculated from all the items. Higher scores indicated a more *distal* approach to infant care. The alpha coefficient of the scale in this sample of mothers was 0.80.

DOL was also assessed at 1 week using the question: ‘When did your milk come in?’ The number of days was then calculated using the infant's birth date and was classified as a dichotomous variable: *on‐time*—within 3 days of the infant's birth; or *delayed*—4 or more days since the infant's birth. However, this question was only included after the study had begun, resulting in missing data on this variable for roughly one‐third of our study sample.

We measured maternal self‐efficacy following the methodology described by Olioff and Aboud (1991). Each woman listed 10 specific activities she believed characterized good mothering. She was then asked how confident she was in her own ability to perform the activities she listed on a 6‐point scale ranging from 1 (*I'm unable do this*) to 6 (*I'm perfectly able to do this*). A score was then calculated by adding all the responses and a percentage was obtained based on a maximum total score of 60 points for 10 items.

Number of breast feeds was obtained from 3‐day infant feeding recalls. Average daily percent weight change in the infant was assessed by calculating the percent difference in measurements taken divided by the number of days between each visit.

### 2.3 Hypotheses

1. Our hypotheses related to crying and PIM were that:
  1. One or more of our four crying variables would be cross‐sectionally associated with report of PIM in the first week of breastfeeding (i.e., at 1 week of age);
  2. One or more of our four crying variables would be longitudinally associated with the report of PIM.

2. Our hypotheses related to crying and LPS were that:
  1. One or more of our four crying variables would be cross‐sectionally associated with LPS at 1 week of age;
  2. Crying at one time‐point would be associated with a change in LPS from one visit to the next.

**FIGURE 1:**
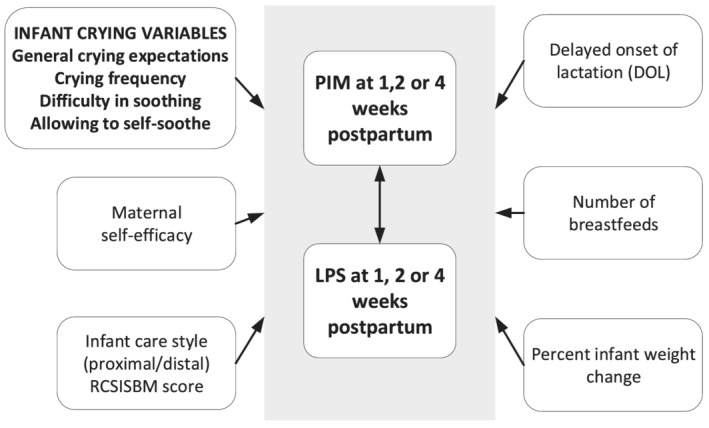
Hypothesized associations between crying and perceived insufficient milk (PIM) and between crying and lactation problem score (LPS)

We also hypothesized that associations between crying and PIM or crying and LPS would be affected by other variables such as DOL, number of breastfeeds, average daily percent weight change the infant care style mothers most agreed with (*proximal* or *distal*) and/or maternal self‐efficacy (Figure 1).

### 2.4 Statistical analysis

For the cross‐sectional analyses we only included mothers fully breastfeeding (*N* = 230) at visit 1 (Hypotheses 1.1 and 2.1). For the longitudinal analyses (Hypotheses 1.2 and 2.2), we limited the sample to those fully breastfeeding at the previous visit (*N* = 230 at week 1 and *N* = 244 at week 2).

We used four different models to test our hypotheses. For the hypotheses related to crying and PIM we fit a logistic regression (Hypothesis 1.1) and a logistic multilevel model (Hypothesis 1.2). For the hypotheses related to crying and LPS, we fit an ordinal logistic regression (Hypothesis 2.1) and a generalized ordered logit/partial proportional odds model (gologit2) (R. Williams, 2006, 2016; R. Q. Williams & Christopher, 2019) (Hypothesis 2.2). Details of the models are presented in the Supporting Information. STATA version 15.2 was used for all analyses.

Each of the models included the crying variables (i.e., general crying expectations, crying frequency, difficulty in soothing, allowing to self‐soothe) as well as other predictor variables potentially associated with PIM or LPS (DOL, number of breastfeeds, average daily percent infant weight change, RCSISBM score and visit). RCSISBM score and number of breastfeeds were conceptualized as confounding variables associated both with crying variables and with PIM. We decided to adjust for potential confounders including maternal education, age, mode of delivery and maternal self‐efficacy as these have been found to be associated with exclusive and predominant breastfeeding (Gonzalez de Cossio, Escobar‐Zaragoza, Gonzalez‐Castell, Reyes‐Vazquez, & Rivera‐Dommarco, 2013; Prior et al., 2012) and/or the report of PIM (McCarter‐Spaulding & Kearney, 2001; Segura‐Millan, Dewey, & Perez‐Escamilla, 1994). However, these were not found to be significantly associated with PIM at 1 week (see Table 1) and were not included in the models. Missing data for any of the variables resulted in the observation being dropped in the fitted model.

**TABLE 1:** Maternal and infant characteristics by report of PIM at first home visit (1 week) among women fully breastfeeding (*N* = 230)

	*N*	No PIM *N* = 200 (87%)	*N*	PIM *N* = 30 (13%)	*P* value^a^
Maternal age, y median [25ile, 75ile]	200	21 [19–24]	30	22 [20–25]	0.341
Maternal BMI, kg/m^2^ median [25ile, 75ile]	191	26.0 [23.6–28.2]	29	26.4 [23.7–28.5]	0.572
Single mother, %	200	5 (2.5%)	30	1 (3.3%)	0.572
At least 12 years education, %	200	99 (49.5%)	30	16 (53.3%)	0.845
Vaginal delivery, %	199	76 (38.2%)	30	15 (50.0%)	0.235
Child gender, % female	200	103 (51.5%)	30	17 (56.7%)	0.696
Gestational age median [25ile, 75ile]	200	39 [38–40]	30	39 [39–40]	0.480
Birth weight, g median [25ile, 75ile]	200	3050 [2812–3350]	30	3088 [2800–3300]	0.558
% wt change from birth median [25ile, 75ile]	194	6.2 [2.3–11.3]	30	4.4 [−2.5–9.6]	0.260
Weight loss >10% of birth weight at 1 week, %	194	3 (1.6%)	30	0 (0.0%)	1.000
Number of breastfeeds median [25ile, 75ile]	200	8 [7,10]	30	9 [7,10]	0.220
LPS median [25ile, 75ile]	196	1 [1,1]	30	2 [2,2]	<0.0001
Onset of lactation, %	200		30		
Not delayed		91 (45.5%)		15 (50.0%)	0.876
Delayed		42 (21.0%)		5 (16.7%)	
Missing		67 (33.5%)		10 (33.3%)	
RCSISBM score, % median [25ile, 75ile]	198	42.7 [35.6–50.0]	29	40.1 [37.8–45.0]	0.393
Maternal self‐efficacy, % median [25ile, 75ile]	197	98.3 [94.4–100.0]	30	98.3 [93.3–100.0]	0.670

Abbreviations: LPS, lactation problem score; PIM, perceived insufficient milk; RCSISBM, responsiveness to crying, schedules and infant satisfaction with breast milk questionnaire.

^a^ *P* values of two sample Wilcoxon ranksum test for continuous variables and Fisher's exact test for categorical variables comparing mothers who reported PIM versus those who did not.

### 2.5 Ethical considerations

Ethical approval was obtained from The Johns Hopkins University Committee on Human Research, the Instituto Mexicano del Seguro Social (IMSS) National Research Commission and the University of Surrey Ethics Committee (CGA Ref No. UEC2019 023FHMS (RC4062)).

## 3 RESULTS

**FIGURE 2:**
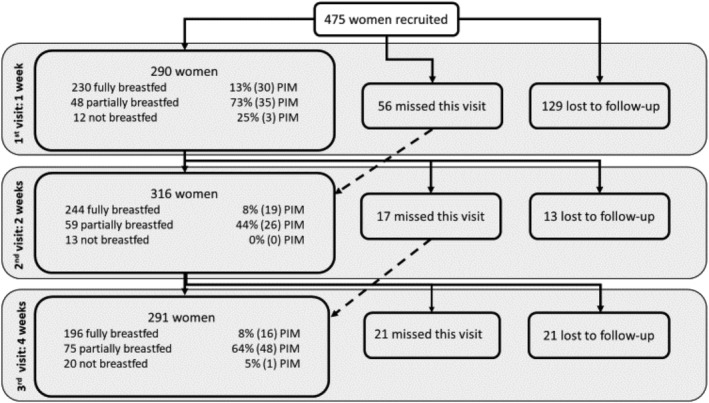
Flow of women recruited to the study

A total of 475 women were recruited to the study. Roughly one‐fourth of the women (26%) were lost‐to‐follow‐up after recruitment (Figure 2). This is likely related to the burden of an hour‐long interview, the high mobility of the population, and other factors related to the large urban environment in which the study took place. The only significant difference between those lost‐to‐follow‐up after recruitment and those with 1 visit completed was a higher proportion of single women in the lost to follow‐up group (9.2% vs. 2.9%).

Two hundred ninety women were visited at 1 week. Twenty‐one percent had already begun adding formula to their infant's diet. The main lactation problems described were PIM (quality—only 1 woman or quantity—52 women, total 53/290 or 19%), painful or cracked nipples (24/290, 8%), inverted nipples (14/290, 5%) and infant sleepy or rejecting the breast (5/290, 2%). Fifteen women reported PIM in other open‐ended questions not directly related to lactation problems. The report of PIM (63% vs. 13%, Fisher's exact *P* < 0.0001) and DOL (57% vs. 31%, Fisher's exact *P* = 0.009) was significantly higher among those not fully breastfeeding than among those who were. LPS was also higher among those not fully breastfeeding (mean [*SD*]: 1.36 [0.75] vs. 2.64 [1.35], Wilcoxon ranksum *P* < 0.0001), but average daily percent weight change and maternal self‐efficacy were not. Among those fully breastfeeding, those who experienced DOL had significantly lower RCSISBM scores (i.e., greater agreement with proximal infant care styles) than those reporting timely onset of lactation (mean [*SD*]: 38.3 [9.2] vs 42.7 [8.3], Wilcoxon ranksum, *P* = 0.0108).

Maternal and infant baseline characteristics of those fully breastfeeding (*N* = 230) are listed in Table 1 by report of PIM at the first week. Comparisons were made of those reporting PIM versus those who did not were made using two sample Wilcoxon ranksum test for continuous variables and Fisher's exact test for categorical variables. No statistically significant differences were found with the exception of LPS, which was significantly higher among those reporting PIM. The median age of the women participating in the study was 21 years, and around 50% had completed at least 12 years of formal education. Over 97% were married or living with a partner and roughly three quarters reported an average household monthly income of two minimum salaries or less (approximately equivalent to US $ 256 per month in the year 2000).

Infants fully breastfed at 1 week and delivered by caesarean had lower percent weight change (i.e., increased weight loss or lower weight gain from birth) in the first 7 days of life than those delivered vaginally (mean [*SD*]: 4.9 [7.6] caesarean vs 8.9 [7.0] vaginal delivery, Wilcoxon ranksum *P* = 0.0002). On the other hand, there was no significant differences by mode of delivery in the percentage of women reporting PIM (11% for caesarean vs 16% vaginal, Fisher's exact *P* = 0.235) or in LPS scores (mean [*SD*] 1.39 [0.83] for caesarean vs 1.33 [0.62] for vaginal, Wilcoxon ranksum *P* = 0.7004) at 1 week.

Reported DOL was significantly associated with lower number of breastfeeds at 1 week in this sample of fully breastfeeding women (mean [*SD*]: 8.0 [1.8] delayed vs 9.4 [2.4] timely onset of lactation, Wilcoxon ranksum *P* = 0.0025). The proportion of women reporting DOL in our sample was also significantly higher among those who delivered by caesarean as compared with those delivering vaginally (41% DOL among caesarean vs 20% DOL among vaginal, Fisher's exact *P* = 0.007).

We hypothesized that crying was associated with PIM both cross‐sectionally (at 1 week, Hypothesis 1.1) and longitudinally (Hypothesis 1.2). We also hypothesized that crying was cross‐sectionally associated with LPS (Hypothesis 2.1) or longitudinally associated with change in LPS (Hypothesis 2.2). Results for each of the four models fit to test these hypotheses are listed in Table 2.

Hypothesis 1.1 Cross‐sectional associations between crying and PIM at 1 week.

**TABLE 2:** Variables associated with the report of PIM and LPS (outcomes) in multivariate analyses

	Cross‐sectional crying and PIM at 1 week PIM (yes/no) (Hypothesis 1.1)		Longitudinal crying and PIM from 1 to 4 weeks PIM (yes/no) (Hypothesis 1.2)		Cross‐sectional crying and LPS at 1 week LPS (6‐point scale: *none* to *too many*) (Hypothesis 2.1)		Longitudinal crying and change in LPS from 1 to 4 weeks LPS (same/increase vs decrease) (Hypothesis 2.2)	
	OR (95% CI)		OR (95% CI)		OR (95% CI)		OR (95% CI)	
Crying predictor variables:								
General crying expectations (6‐point scale: *not at all* to *too much*)	0.94 (0.51–0.71)	0.835	**2.07 (1.37–3.12)**	**<0.0001**	1.01 (0.65–1.59)	0.951	1.36 (0.99–1.86)	0.057
Crying frequency (number of times cried/fussed per day)	1.06 (0.94–1.19)	0.376	1.04 (0.95–1.14)	0.385	**1.12 (1.01–1.23)**	**0.024**	0.99 (0.93–1.07)	0.854
Difficulty in soothing (6‐point scale: *very easily* to *with great difficulty*)	1.17 (0.72–1.91)	0.520	0.79 (0.52–1.22)	0.291	**1.78 (1.24–2.57)**	**0.002**	0.74 (0.51–1.07)	0.110
Allowing to self soothe (6‐point scale: *never* to *always*)	1.36 (0.67–2.73)	0.392	0.79 (0.42–1.48)	0.454	1.47 (0.82–2.64)	0.201	**0.53 (0.32–0.86)** ^a^	**0.011**
Other predictor variables:								
LPS (6‐point scale: *none* to *too many*)	**2.95 (1.47–5.90)**	**0.002**	**1.85 (1.22–2.79)** ^a^	**0.003**	n/a	n/a	**0.026 (0.004–0.138)** ^a^	**>0.0001**
DOL (yes/no)	0.64 (0.16–2.59)	0.563	**2.49 (1.05–5.91)**	**0.039**	0.65 (0.23–1.87)	0.427	**2.22 (1.11–4.41)**	**0.023**
Number of breast feeds (number per day)	1.08 (0.86–1.35)	0.528	1.01 (0.85–1.20)	0.912	1.02 (0.85,1.22)	0.804	**0.85 (0.73–0.98)** ^a^	**0.029**
Average daily % infant weight change (%)	1.06 (0.62–1.82)	0.831	1.15 (0.65–2.05)	0.622	**0.55 (0.35–0.85)**	**0.007**	0.96 (0.62–1.48)	0.862
RCSISBM score (%)	1.00 (0.93–1.07)	0.923	1.01 (0.97–1.06)	0.577	0.95 (0.89–1.00)	0.066	**1.04 (1.01–1.08)**	**0.026**
Visit (weeks)	n/a	n/a	1.21 (0.80–1.83)	0.367	n/a	n/a	0.85 (0.62–1.18)	0.336
Number of observations	148^b^		303 (170 clusters)^b^ ^,^ ^c^		148^b^		296 (168 clusters)^b^ ^,^ ^c^ ^,^ ^d^	
Pseudo *R* ^2^	0.1512				0.1214		0.3433	

Abbreviations: LPS, lactation problem score; PIM, perceived insufficient milk; RCSISBM, responsiveness to crying, schedules and infant satisfaction with breast milk questionnaire.

We used bold emphasis to highlight associations significant at the *p* < 0.05 level.

^a^ Lagged variable.

^b^ Seventy‐seven women were missing data on the onset of lactation and were dropped from the models; the remaining five were missing data on LPS. Daily percent infant weight change and/or RCSISBM score and were also dropped.

^c^ Observations were clustered by woman and not all women contributed observations for all visits; in addition, models excluded observations with missing data and were limited to those fully breastfeeding at the previous visit.

^d^ Two women had missing data on change in LPS.

Crying was not associated with PIM among women fully breastfeeding at 1 week, but LPS was.

Hypothesis 1.2 Longitudinal associations between crying and PIM in the first 4 weeks.

Expecting the infant to cry more in comparison with other infants (i.e., higher general crying expectation score as assessed at recruitment) was associated with higher odds of reporting PIM at 2 or 4 weeks. Higher LPS at the previous visit and DOL were also associated with higher odds of reporting PIM at 2 and 4 weeks. Interestingly, although maternal self‐efficacy was not significantly associated with reports of PIM, both higher general crying expectations (Spearman's rho: −0.16, *P* = 0.015) and lower LPS at 1 week (Spearman's rho: −0.15, *P* = 0.028) were also each negatively associated with maternal self‐efficacy in univariate analyses.

Hypothesis 2.1 Cross‐sectional associations between crying and LPS at 1 week.

Both higher crying frequency and greater difficulty in soothing the infant were associated with greater odds of reporting a higher LPS at 1 week. Greater daily percent infant weight change was associated with lower odds of reporting high LPS at 1 week.

Hypothesis 2.2 Longitudinal associations between crying and change in LPS from 1 to 2 weeks and 2 to 4 weeks.

Allowing the infant to self‐soothe more frequently at the previous visit was associated with lower odds of LPS remaining the same or increasing from 1 to 2 weeks or 2 to 4 weeks, as were higher LPS and higher number of breastfeeds at the previous visit. Women reporting DOL had higher odds of LPS staying the same or increasing as compared with those reporting timely onset of lactation. In addition, a unit increase in percent RCSISBM score (i.e., a more *distal* infant care style) was associated with higher odds of LPS staying the same or increasing.

## 4 DISCUSSION

The first week postpartum is an important time for the initial establishment of lactation (Brownell, Howard, Lawrence, & Dozier, 2012; Galipeau, Goulet, & Chagnon, 2012; Matias, Nommsen‐Rivers, Creed‐Kanashiro, & Dewey, 2010; Perez‐Escamilla, Buccini, Segura‐Perez, & Piwoz, 2019). We confirmed that PIM (DaMota, Banuelos, Goldbronn, Vera‐Beccera, & Heinig, 2012), DOL (Segura‐Millan, Dewey, & Perez‐Escamilla, 1994) and lactation problems (Babakazo, Donnen, Akilimali, Ali, & Okitolonda, 2015; Gianni et al., 2019) were associated with the addition of formula to the infant's diet.

For 79% of women fully breastfeeding at 1 week, we sought to identify whether crying and other variables such as LPS, DOL, breastfeeding frequency and/or infant care style were associated with the report of PIM. We found a statistically significant association between our LPS measure and PIM at 1 week. Lactation problems including nipple soreness and infant latching problems can lead to inadequate and/or infrequent removal of milk from the breast, lowering milk production in the fine‐tuned demand and supply process that characterizes breastfeeding (Neifert, 2004). Previous literature also suggested that factors such as infant crying (Sacco, Caulfield, Gittelsohn, & Martinez, 2006; Segura‐Millan, Dewey, & Perez‐Escamilla, 1994), DOL (Perez‐Escamilla, Buccini, Segura‐Perez, & Piwoz, 2019; Segura‐Millan, Dewey, & Perez‐Escamilla, 1994) and number of breastfeeds (Galipeau, Dumas, & Lepage, 2017) were likely to be significantly associated with PIM, but this was not the case for our data. We considered that women who experienced the initial hurdles of DOL and/or PIM and were not giving any formula at 1 week may have been more committed to breastfeeding their infant. This is supported by our finding that, among those fully breastfeeding a 1 week, those reporting DOL had significantly lower RCSISBM scores (i.e., greater agreement with proximal infant care styles) than those reporting timely onset of lactation.

Longitudinally examining the factors related to PIM at 2 or 4 weeks confirmed the association between LPS and PIM. Odds of reporting PIM at 2 or 4 weeks were greater for those reporting a higher LPS at the previous visit, providing some evidence that initial breastfeeding problems precede reports of PIM and consequent introduction of formula.

We found that higher general crying expectations were also longitudinally associated with report of PIM at 2 and 4 weeks. One explanation is that this as an indication of lower parenting self‐efficacy. Greater parenting self‐efficacy has been linked with better postnatal adjustment (Mihelic, Filus, & Morawaska, 2016). Although our measure of self‐efficacy was not significantly associated with report of PIM, it was negatively associated with general crying expectations. Thus, the mother's expectation that her infant will cry more may be a sign of lower self‐efficacy in other areas of parenting, including her ability to soothe her infant when crying or produce enough breast milk. Alternatively, higher general crying expectations may also be associated with a perception that breastfed infants will cry more, and that formula fed infants are more settled (Brown, Raynor, & Lee, 2011). In our sample, reported crying frequency was not significantly different between infants who were fully breastfed and those who were also given formula (results not shown).

Primiparous mothers have reported feeling unprepared for the early postpartum period and not having realistic expectations of their infant's behaviour (DaMota, Banuelos, Goldbronn, Vera‐Beccera, & Heinig, 2012). In fact, it has been previously suggested that better maternal understanding and expectations of normal patterns of infant behaviour would lead to fewer lactation problems (Brown, Rance, & Bennett, 2016). Our findings related to the significant positive associations between crying variables (crying frequency, difficulty in soothing) and LPS at 1 week are in line with these observations. The limited gastric capacity of very young infants results in their need to be fed frequently, often signalled by crying. First‐time mothers may be surprised and worried by this need to be fed at such short intervals, prompting them to report higher LPS. Infant crying is also the main means an infant has to signal a variety unmet needs (e.g., physical discomfort, insecurity, tiredness). Some mothers may overinterpret their infant's cries as hunger and, if they are not meeting their infant's other needs, view the challenges they are facing in soothing their infant as a sign of greater breastfeeding difficulties (i.e., higher LPS). On the other hand, it is also possible that some of these infants were not receiving adequate amounts of breastmilk in this first week. This is supported in part by the significant association between lower percent weight change (i.e., increased weight loss or lower weight gain from birth) at 1 week and higher LPS. Additional breastfeeding support is needed for mothers in the early postpartum period, especially among those whose infants are losing more weight (Kelly, Keane, Gallimore, Bick, & Tribe, 2020). Decisions to introduce formula often occur during the postpartum hospital stay (Biggs et al., 2018), and introduction is associated with increased risk of infections (Duijts, Ramadhani, & Moll, 2009) and early abandonment of breastfeeding (Chantry, Dewey, Peerson, Wagner, & Nommsen‐Rivers, 2014). Others have also reported that breastfeeding difficulties are a significant predictor of in hospital decisions to introduce formula (Bentley et al., 2017) and that a greater proportion of mothers whose infants had early weight loss >10% reported concern about their milk supply at 2 weeks (42% vs 20%, respectively) (Flaherman, Beiler, Cabana, & Paul, 2016).

A recent systematic review suggested that a higher risk of increased weight loss was evident among infants born by caesarean, although it is still not clear if this early initial weight loss negatively impacts future infant health (Kelly, Keane, Gallimore, Bick, & Tribe, 2020). Matias, Nommsen‐Rivers, Creed‐Kanashiro, and Dewey (2010) also reported that excess neonatal weight loss (10% or more of birth weight by day 3) was significantly associated with caesarean delivery, but not with DOL. Mexico is a country with one of the highest proportions of caesarean deliveries—caesarean deliveries were reported as being around 55% of all live births at IMSS facilities in Mexico City in 2008, which decreased significantly to 48% of all live births in 2017 (Uribe‐Leitz et al., 2019). In our sample the percentage of infants delivered by caesarean was 60%, with greater weight loss among these infants than among those delivered vaginally. Caesarean delivery has been associated with significantly lower early breastfeeding rates (any initiation or at hospital discharge) (Prior et al., 2012) as well as later initiation of breastfeeding (Takahashi et al., 2017) and greater likelihood of being given formula within 48 h of delivery (Matias, Nommsen‐Rivers, Creed‐Kanashiro, & Dewey, 2010). Giving birth by caesarean was not significantly associated with LPS in our sample. This may be explained by our inclusion here only of women fully breastfeeding at 1 week, and thus, women who had given birth by caesarean and were still fully breastfeeding may have experienced fewer early lactation problems. Change in LPS over time was significantly associated with 5 of our explanatory variables, including infant care styles (i.e., distal: less physical contact, scheduled feedings, leaving the baby to cry or proximal: more physical contact, on demand feeding, response to crying). Brown and Arnott (2014) found that increased use of parent‐led routines and low levels of nurturance (including physical contact with the baby) were associated with formula use at birth or short breastfeeding duration, whereas an approach led by infant cues was associated with longer breastfeeding duration. St James‐Roberts et al. (2006) reported that parents who promptly responded to crying and had extended physical contact with their infant breastfed more frequently and for longer; Little, Legare, and Carver (2018) found that increased physical contact predicted feeding responsiveness (i.e., feeding in response to early infant hunger cues rather than waiting for the infant to cry) and greater self‐reported feeding responsiveness was associated with more frequent breastfeeding throughout the day and greater likelihood of exclusive breastfeeding; Iacovou and Sevilla (2013) reported that feeding to schedule was associated with lower breastfeeding rates and shorter breastfeeding duration. In our sample, a higher RCSISBM score (i.e., a more distal infant care style) was significantly associated with higher odds of LPS remaining the same or increasing in the first 4 weeks. Our RCSISBM measure also included specific statements related to whether an infant could be satisfied with breast milk alone. With an alpha coefficient of 0.80 for the RCSISBM in this sample, we provide further evidence of a link between general distal infant care styles and greater alignment with the belief that breast milk alone is insufficient to satisfy an infant.

Although we found that allowing the infant to self‐soothe more often than *hardly ever* was associated with lower odds of stable or higher LPS, this was unexpected. Only 6% of women reported adopting this practice and it may be a spurious finding. Alternatively, this small proportion of infants may have an easy‐going nature, with few breastfeeding problems and difficulties in soothing themselves, resulting in their mothers allowing them to self‐soothe more often (Harries & Brown, 2019). This variable may be also capturing a different aspect of maternally reported responses to crying unrelated to breastfeeding which may warrant further exploration.

DOL is one of the early lactation problems experienced by new mothers and has been associated with lower breastfeeding frequency (Galipeau, Goulet, & Chagnon, 2012). We also found that DOL was significantly associated with lower number of breastfeeds at 1 week. In addition, DOL was associated with higher odds of stable or increasing LPS, whereas higher breastfeeding frequency was associated with lower odds of stable or increasing LPS. The proportion of women reporting DOL in our sample (33%) was comparable with primiparous women in another study (34%) (Dewey, Nommsen‐Rivers, Heinig, & Cohen, 2003), and we also found it to be significantly higher among those not delivering vaginally as reported by others (Brownell, Howard, Lawrence, & Dozier, 2012).

Thus, although most study participants reported low initial LPS, factors such as DOL, not breastfeeding as often, and greater agreement with a distal infant care style led their LPS to remain stable or increase. Women who started out with a higher LPS tended to report a decrease in LPS, even more so if their milk came‐in within 3 days postpartum, breastfeeding was more frequent, and they reported greater agreement with the proximal infant care style.

In this study we provide evidence that early lactation problems among women fully breastfeeding precede later reports of PIM. Our study underscores the need for lactation support in the early postpartum weeks especially among women experiencing DOL (Brownell, Howard, Lawrence, & Dozier, 2012; Galipeau, Goulet, & Chagnon, 2012; Matias, Nommsen‐Rivers, Creed‐Kanashiro, & Dewey, 2010; Perez‐Escamilla, Buccini, Segura‐Perez, & Piwoz, 2019). Mothers' perception of increased breastfeeding frequency must shift from a sign of PIM (Kent, Prime, & Garbin, 2012) to a key approach to increasing the volume of breast milk produced, as our findings suggest that perceived lactation problems can diminish with increased breastfeeding frequency. We also provided evidence that decreasing lactation problems are associated with greater alignment with the proximal infant care style. Proximal infant care styles have the potential of not only improving breastfeeding outcomes but also reducing infant crying (St James‐Roberts et al., 2006), promoting better maternal–infant interactions and improving child IQ at later ages (Iacovou & Sevilla, 2013). Finally, increasing maternal confidence in their ability to soothe their infant when crying may also have implications related to lactation problems reported by new mothers.

Our study is the first, to our knowledge, to examine longitudinally variables related to PIM in the first month of life among fully breastfed infants. We also provided evidence of a correlation between agreement with distal approaches to infant care and concurrence that breast milk alone is not enough to satisfy an infant. Its limitations include the relative absence of prenatal measures of maternal alignment with distal or proximal infant care styles or breastfeeding self‐efficacy; a lack of information on key variables between birth and the first week visit (i.e., timing of breastfeeding initiation, rooming‐in, use of prelacteal feeds, pacifiers or nipple shields, infant crying behaviour) and the absence of data on the onset of lactation for the entire sample. The sample characteristics of this population were also relatively homogeneous (mid‐to‐lower class first‐time Mexican mothers) and may be of limited generalizability to other populations. On the other hand, PIM is a world‐wide phenomenon, and similar associations between infant care styles and breastfeeding have also been observed in different countries.

There is a need for future research to explore if interventions providing women with anticipatory guidance on normal infant crying behaviour, the pros and cons of adopting a more proximal or distal infant care style, as well as challenges which they may be unfamiliar with but which are often encountered in the first days of breastfeeding (such as initial nipple pain or the DOL) would result in fewer lactation problems, a decrease in the number of women reporting PIM and an increase in the number of women exclusive breastfeeding.

## CONFLICTS OF INTEREST

The authors declare that they have no conflicts of interest.

## CONTRIBUTIONS

LM and LC designed the study. LM conducted the study with support from LC and HM. LM analysed the data and drafted the manuscript with support from PH, SB, MR and MMR. All authors (LM, SB, MPR, MMR, HM, LC) revised the manuscript and approved its final version. LM has primary responsibility for the final content.

## Supporting information


**Data S1** Supporting Information
