# Supplementary material for: Perceived insufficient milk among primiparous, fully breastfeeding women: Is infant crying important?
Source: Matern Child Nutr. 2021 Jan 5;17(3):e13133. doi: 10.1111/mcn.13133 (PMC8189230; doi:10.1111/mcn.13133)
Supplement: Supplementary file 1 — Data S1 Supporting Information [file MCN-17-e13133-s001.docx]

**Supplementary Material**

Final fitted models used to test each hypothesis are as follows:

**Analysis for Hypothesis 1.1**: A logistic regression model to examine the effects of crying variables on report of PIM (yes=1/no=0) at 1 week:

*Log odds of reporting PIM at 1 week (Yi=1) = β_0_* *+* *β_1_General crying expectations + β_2_Crying frequency + β_3_Difficulty in soothing + β_4_Allowing to self-soothe + β_5_LPS + β_6_DOL + β_7_Number of breast feeds + β_8_Average daily percent infant weight change + β_9_RCSISBM score* *+ ɛ_i_*

**Analysis for Hypothesis 1.2**: A logistic multilevel model to examine the lag effect of crying variables on the report of PIM (yes=1/no=0) in the first 4 weeks of life:

*Log odds of reporting PIM in the first 4 weeks (Yi=1) = β_0_ + β_1_General crying expectations + β_2_Crying frequency + β_3_Difficulty in soothing + β_4_Allowing to self-soothe + β_5_LagLPS + β_6_DOL + β_7_Number of breast feeds + β_8_Average daily percent infant weight change +* *β_9_RCSISBM score + β_10_Visit + µ_0j_ + ɛ_i_*

**Analysis for Hypothesis 2.1**: An ordinal logistic regression to examine the effects of crying variables on LPS (1= *none* to 6=*too many)*:

*Log odds of moving from a lower to a higher LPS at 1 week (Yi>j) = β_0_ + (β_1_General crying expectations - к_j_) + (β_2_Crying frequency - к_j_) + (β_3_Difficulty in soothing- к_j_)* *+ (β_4_Allowing to self-soothe - к_j_) + (β_5_DOL-* *к_j_) + (β_6_Number of breast feeds - к_j_) + (β_7_Average daily percent infant weight change - к_j_) + (β_8_RCSISBM score - к_j_) + ɛ_i_*

**Analysis for Hypothesis 2.2:** A generalized ordered logit/ partial proportional odds model for ordinal dependent variables (gologit2) (R. Williams, 2006, 2016; R. Q. Williams, Christopher, 2019) to analyse the lag effect of crying variables on the change in LPS over time (1=*decreased*, 2=*remained the same*, 3=increased).

*Log odds that LPS problems will remain the same/increase (Yi>1) = α_1_ + β_1_General crying expectations + β_2_LagCrying frequency + β_3_LagDifficulty in soothing + β_4_LagAllowing to self-soothe + β_1_LagLPS + β_2_DOL + β_3_Lag Number of breast feeds + β_4_Average daily percent infant weight change + β_5_RCSISBM score + β_6_Visit + ɛ_i_*

**Visual summary of results for each hypothesis:**

**Hypothesis 1.1: Cross-sectional association between crying and PIM at 1 week: rejected**

**Hypothesis 1.2: Longitudinal association between crying and PIM in the first 4 weeks: confirmed**

**Hypothesis 2.1: Cross-sectional association between crying and LPS in the first week: confirmed**

**Hypothesis 2.2: Longitudinal association between crying and change in LPS in the first 4 weeks: confirmed**

**Responsiveness to Crying, Schedules and Infant Satisfaction with Breast Milk questionnaire (RCSISBM) in Spanish**

**Percepción Materna del Llanto**

***Para las siguientes preguntas considere solamente bebés menores de 6 meses****.*

Utilizando la siguiente escala, por favor indique el cuadro que mejor describe lo que piensa usted acerca de estas afirmaciones, tomando en cuenta lo siguiente:

1. No hay verdadero ni falso, tampoco respuestas correctas o incorrectas, sólo una descripción adecuada de su bebé.
2. Por favor califique tomando en cuenta el comportamiento actual y reciente de su bebé (en el último mes).
3. Conteste cada pregunta por separado. Algunas preguntas pueden parecer iguales, pero no lo son. No conteste a todas las preguntas similares de la misma manera intencionalmente.
4. Utilice los extremos de la escala siempre que sea apropiado. Trate de evitar usar con mucha frecuencia los puntos medios de la escala.
5. Conteste cada pregunta pronto. Si no puede decidir, siga adelante con la próxima y luego regrese a esta pregunta después.
6. Trate de contestar todas las preguntas. Deje sin contestar solamente aquellas para las cuales no tenga información o que no se aplican a su bebé.
7. Considere únicamente sus propias impresiones y observaciones del bebé.

| 1 | 2 | 3 | 4 | 5 | 6 |
| --- | --- | --- | --- | --- | --- |
| Completamente en desacuerdo | En  desacuerdo | Ligeramente en desacuerdo | Ligeramente de acuerdo | De acuerdo | Completamente de acuerdo |

**Llanto como señal**

1. El llanto es la única manera que tiene un bebé para comunicarse.
2. El bebé puede indicar lo que quiere por medio de movimientos y gestos, sin hacer ruidos.
3. Si el bebé no llora, la mamá no se va a dar cuenta que el bebé necesita algo.
4. Un bebé puede pedir comida a su mamá hablándole con palabras.
5. El llanto es la única manera de expresarse del bebé.
6. El bebé puede indicar, sin llorar, que tiene hambre.
7. El bebé puede expresarse por medio de movimientos y gestos, sin hacer ruidos.
8. El llanto es la única manera que tiene un bebé para que le hagan caso.
9. Un bebé no necesita pedir las cosas – él las puede hacer y conseguir por sí mismo.
10. El bebé no puede comunicar que tiene hambre sin llorar.

**El bebé y su llanto**

11. El bebé debe comer solamente a sus horas.

12. Hay que dejar el bebé llorar solito de vez en cuando para que se acostumbre a estar solo.

13. Cuando un bebé llora, siempre hay que atenderle pronto, ver que tiene y sanar su necesidad.

14. El bebé tiene que saber aguantar su llanto cuando la mamá está ocupada.

15. Si siempre se le hace caso al bebé cuando está llorando lo están consintiendo demasiado.

16. Cuando el bebé quiere algo y uno no puede dárselo, hay que dejarlo llorar solito, para que aprenda que la vida no es fácil.

17. Hay que dejar el bebé llorar solito de vez en cuando para que sea independiente.

18. Si un bebé llora mucho por naturaleza, hay que tenerle paciencia y buscar satisfacer sus necesidades antes de que empiece a llorar.

19. El bebé puede escoger esperar pacientemente a que la mamá lo atienda, o entonces hacer un berrinche.

20. Cuando el bebé quiere algo y uno no puede dárselo, hay que distraerlo hablándole, cantándole, dándole un juguete o haciendo otra cosa.

21. No hay que atender el bebé de inmediato cuando llore, para que aprenda a tener paciencia.

22. Un bebé que llora frecuentemente es un bebé que quiere todo a tiempo.

23. La necesidad que tiene un bebé de ser cargado y de cariño es tan importante cuanto la de comer, dormir o estar limpio.

24. La razón principal por la cual llora un bebé es por no asistir a su programa favorito en la televisión.

25. El bebé no puede escoger cuanto tiempo va a llorar – él llora hasta que la necesidad que tenga sea satisfecha.

**El llanto y la alimentación del bebé**

26. Para que el bebé quede satisfecho, hay que darle pecho y leche de fórmula.

27. Para que el bebé menor de 6 meses se llene bien, hay que añadir cosas a la leche materna.

28. Cuando el bebé quiere seguir chupando el pecho aunque no salga leche es porque le gusta chupar las cosas (el pecho, la mano, el chupón) y estar cerca de su mamá.

29. Cuando un bebé llora muy seguido por la noche es porque no le llena el pecho.

30. Para que el bebé no tenga sed, hay que darle sólo pecho a libre demanda.

31. Cuando el bebé quiere seguir chupando el pecho aunque no salga leche es porque no se da cuenta que ya no hay leche.

32. Para que el bebé quede satisfecho, hay que darle un guisado picante y muy condimentado.

33. Se le debe dar de comer al bebé cada vez que le pida, aunque no le toque.

34. Para que el bebé no tenga sed, hay que darle agua y té.

35. Para que el bebé quede satisfecho, hay que darle pecho y algún jugo de frutas.

36. Si le acaban de dar de comer y el bebé sigue llorando, es porque le faltó el postre.

37. La leche materna no es suficiente para el bebé si le acaban de dar de comer y él sigue llorando.

38. Para que el bebé quede satisfecho, hay que darle sólo pecho a libre demanda.

39. Cuando un bebé pide de comer muy seguido es porque tiene una lombriz y por eso no hay que darle de comer hasta que se muera la lombriz de hambre.
